# Supplementary material for: Transcriptomic Insights into Mechanisms of Early Seed Maturation in the Garden Pea (Pisum sativum L.)
Source: Cells. 2020 Mar 23;9(3):779. doi: 10.3390/cells9030779 (PMC7140803; doi:10.3390/cells9030779)

Zhongwan−6,25(4)

Global Summary

%DE = 0.23

genes with fdr < 0.2 = 466 (223 + / 243 −)

genes with fdr < 0.1 = 423 (205 + / 218 −)

genes with fdr < 0.05 = 389 (184 + / 205 −)

genes with fdr < 0.01 = 324 (155 + / 169 −)

genes in genesets = 0

<FC> = 0

<t−score> = 0

<p−value> = 0.01

<fdr> = 0.77

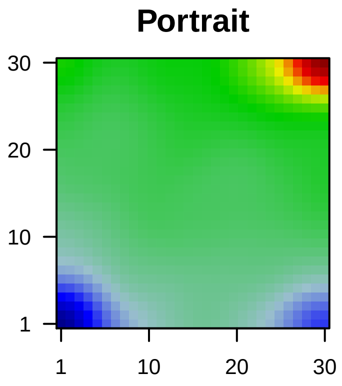

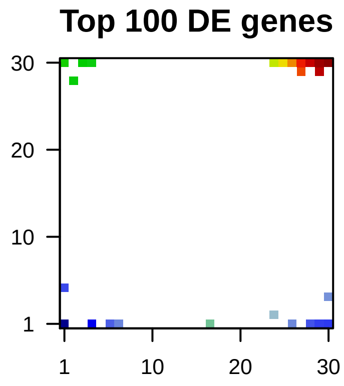

Global Genelist

Rank	ID	log(FC)	p−value	fdr	Metagene	Description
1	TRINITY_DN2	−0.55	2e−16	3e−15	1 x 1	
2	TRINITY_DN9	1.8	2e−16	3e−15	30 x 30	
3	TRINITY_DN7	−0.46	2e−16	3e−15	30 x 30	
4	TRINITY_DN5	2.48	2e−16	3e−15	30 x 30	
5	TRINITY_DN5	0.59	2e−16	3e−15	1 x 1	
6	TRINITY_DN7	0.47	2e−16	3e−15	30 x 30	
7	TRINITY_DN7	−1.12	2e−16	3e−15	1 x 1	
8	TRINITY_DN4	−2.79	2e−16	3e−15	1 x 1	
9	TRINITY_DN9	−0.46	2e−16	3e−15	4 x 30	
10	TRINITY_DN9	−0.8	2e−16	3e−15	30 x 1	
11	TRINITY_DN9	0.74	2e−16	3e−15	30 x 30	
12	TRINITY_DN2	0.62	2e−16	3e−15	29 x 30	
13	TRINITY_DN2	−0.71	2e−16	3e−15	1 x 1	
14	TRINITY_DN2	−0.65	2e−16	3e−15	30 x 1	
15	TRINITY_DN2	1.66	2e−16	3e−15	30 x 30	
16	TRINITY_DN2	−0.51	2e−16	3e−15	1 x 30	
17	TRINITY_DN2	−0.73	2e−16	3e−15	1 x 1	
18	TRINITY_DN2	0.5	2e−16	3e−15	1 x 1	
19	TRINITY_DN3	−0.62	2e−16	3e−15	28 x 1	
20	TRINITY_DN3	−1.81	2e−16	3e−15	1 x 1	

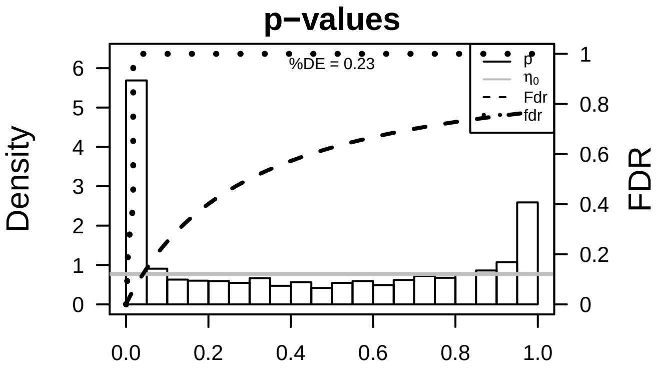

Supplement: Supplementary file 1 [file cells-09-00779-s001.zip › Supplementary materials/FileS4.zip!/FileS3/TFs - Results/Summary Sheets - Samples/Zhongwan.6.25.4..pdf]
